# Prognostic Relevance of Global Myocardial Work Index in Patients with Moderate Aortic Valve Stenosis

**DOI:** 10.3390/jcm12247694

**Published:** 2023-12-14

**Authors:** Joscha Kandels, Michael Metze, Andreas Hagendorff, Stephan Stöbe

**Affiliations:** Department of Cardiology, Leipzig University Hospital, Liebigstr. 20, 04103 Leipzig, Germany; michael.metze@medizin.uni-leipzig.de (M.M.); andreas.hagendorff@medizin.uni-leipzig.de (A.H.); stephan.stoebe@medizin.uni-leipzig.de (S.S.)

**Keywords:** transthoracic echocardiography, moderate aortic valve stenosis, left ventricular deformation, work index, prognostic value

## Abstract

Background: A reduced global myocardial work index (GWI) ≤ 1951 mmHg% is associated with increased mortality in patients with severe aortic valve stenosis (AS). However, parameters predicting the outcome in patients with moderate AS are limited. Therefore, the aim of this study was to evaluate the prognostic value of the GWI in patients with moderate AS. Methods and Results: In this prospective study, 103 patients with moderate AS (mean age 72 ± 10 years; male: 69%) underwent standardized transthoracic echocardiography. The primary endpoint was survival without an aortic valve replacement (AVR). After a median follow-up of 30 ± 5 months, 37 patients (36%) were referred for an AVR. Survival without an AVR was 96% at 12 months and 80% at 30 months (>1951 mmHg%) versus 96% and 68% (≤1951 mmHg%). A GWI ≤ 1951 mmHg% did not predict the need for an AVR (hazard ratio 1.31 (95% CI, 0.63–2.72), *p* = 0.49). Furthermore, there was no significant correlation between the mean GWI (1644 ± 448 mmHg%) and mean aortic valve pressure gradient (24.2 mmHg ± 6.2, *p* = 0.615) or effective aortic orifice area (1.24 cm^2^ ± 0.11, *p* = 0.678). There was no difference between the AVR and non-AVR groups in the occurrence of clinical symptoms. Conclusion: In contrast to patients with severe AS, a GWI ≤ 1951 mmHg% did not predict the need for an AVR. Further research is needed to improve the risk stratification in patients with moderate AS.

## 1. Introduction

Aortic stenosis (AS) is defined as a narrowing of the aortic valve area, which is associated with an increased left ventricular (LV) pressure load. According to the current guidelines, AS is defined by the effective orifice area (EOA), maximum transvalvular flow velocity (Vmax) and mean transvalvular pressure gradient (Pmean) [1]. The optimal timing for an aortic valve replacement (AVR) depends on the presence of symptoms and the guideline criteria [1].

The poor long-term survival of patients with severe AS without an AVR has been well described [2,3]. However, moderate AS has also been shown to be associated with a worse outcome [4]. Unfortunately, the guideline criteria such as the Vmax, Pmean and aortic valve calcification cannot consistently predict the clinical outcome in these patients [3,5,6,7].

Recently, the global myocardial work index (GWI) has gained importance as an echocardiographic parameter to assess LV function. The GWI is based on pressure–strain loops and combines myocardial deformation imaging assessed by 2D speckle tracking with a non-invasive afterload using brachial cuff blood pressure [8]. As the GWI has been shown to be independent from the afterload and related to myocardial deformation and contractile function, this echocardiographic parameter provides an accurate assessment of cardiac performance in respect to the LV load condition. The GWI has been reported to be a useful diagnostic tool in patients with severe aortic stenosis [9,10]. In a previous study by Ilardi et al., a GWI ≤ 1951 mmHg% was associated with a higher mortality in moderate-to-severe AS [11]. In addition, the GWI showed an independent association with symptoms in patients with severe AS [12].

The current data on the progression of moderate AS are limited and the study populations are generally small [13]. The optimal timing for an AVR in moderate AS has not yet been defined. The only recommendations for these patients are the optimal management of comorbidities and risk factors and regular monitoring with transthoracic echocardiography (TTE).

The aim of this study was to evaluate the prognostic value of the GWI in relation to the need for an AVR in patients with moderate AS.

## 2. Materials and Methods

### 2.1. Study Population

This prospective study was conducted in accordance with the Declaration of Helsinki and was approved by the Ethics Committee of the University of Leipzig (041/19-ek). All the enrolled patients gave informed consent. The patients with moderate AS defined by an EOA between 1.0 cm^2^ and 1.5 cm^2^ who underwent TTE between 2016 and 2018 at the University Hospital Leipzig were included. The exclusion criteria were concomitant moderate or severe valvular heart disease, cardiac amyloidosis, hypertrophic obstructive cardiomyopathy, acute myocarditis, an LV ejection fraction (EF) < 45% or/and LV stroke volume index (SVi) < 35 mL/m^2^, pulmonary hypertension due to chronic pulmonary disease and/or acute pulmonary embolism, a body mass index ≥ 35 kg/m^2^ and/or previous heart surgery or valvular intervention.

### 2.2. Follow-Up

A complete follow-up data set was available for 103 of 157 patients. Sixteen patients died, fifteen patients had missing blood pressure values at the time of the TTE, six patients had various significantly premature beats without the possibility of speckle tracking, seven patients had poor image quality and ten patients were lost to follow-up. The patients enrolled in this study were followed clinically and underwent TTE every 6 months for 3 years, with the first TTE performed at the time of enrolment. Transesophageal echocardiography (TEE) was performed for the final assessment if classified as moderate to severe. The patient characteristics were collected from the medical records. At baseline, all the patients were clinically asymptomatic or presented with non-specific and/or only mild symptoms. The primary endpoint was survival without an AVR.

### 2.3. Echocardiography

Transthoracic echocardiography was performed using a Vivid E9 or E95 ultrasound system with an M5-S or a 4 Vc phased array probe, and the echocardiographic analyses were performed with the EchoPac software version 203 (GE Healthcare Vingmed Ultrasound AS, Horten, Norway).

### 2.4. Assessment of Aortic Valve Stenosis

In all the patients, the EOA was calculated using the continuity equation. Therefore, the diameter of the left ventricular outflow tract (D_LVOT_) was measured in the parasternal long-axis view during mid-systole. The left ventricular outflow tract blood flow velocity was measured by pulsed-wave (pw) Doppler in the apical long-axis view at the same position where the D_LVOT_ was assessed. The Vmax was determined in the apical long-axis view by continuous-wave (cw) Doppler across the AV. Based on the flow velocities across the AV, the Pmean was calculated by using a simplified Bernoulli equation (when the pre-stenotic velocities were within normal ranges). The progression from moderate to severe AS was assessed by using the EOA, Vmax and Pmean. All the measurements were performed by experienced cardiologists according to the current recommendations [1,14].

### 2.5. Left Ventricular Function and Morphology

The LV volumes and ejection fraction (LVEF) were assessed by using LV planimetry using the modified Simpson’s rule [15]. According to the current recommendations, a relevant diastolic dysfunction was defined by E/E’ values ≥ 14 in patients in sinus rhythm and ≥11 in patients with atrial fibrillation [16]. A parasternal short-axis M-Mode sweep was performed for the assessment of the LV diameters. LV hypertrophy (LVH) was defined by an LV mass index (LVMi) ≥ 95 g/m^2^ in women and ≥115 g/m^2^ in men. To estimate the right ventricular load, the maximum tricuspid regurgitation velocity (TR_Vmax_) was measured by cw Doppler and defined as pathological ≥ 2.8 m/s [16].

### 2.6. Left Ventricular Deformation

The global longitudinal strain (GLS) was assessed by 2D speckle-tracking analyses (long-axis, 2-chamber, and 4-chamber views) according to the current recommendations [17]. The region of interest (ROI) of the tracking area was individually adjusted to the endocardial and epicardial border to enable the exact measurements [17]. The measurements were only accepted if all the segments had been tracked accurately.

The left ventricular GWI was assessed by the estimated area of the LV pressure–strain loop calculated by the post-processing software EchoPAC version 206, revision 58 (GE-Healthcare, Chicago, IL, USA) and incorporating the patients’ non-invasively measured blood pressure values [18]. The blood pressure measurements were performed with the patients in the supine position at the time of the TTE using an automated arm-cuff blood pressure monitor.

Based on the GWI, other parameters such as the global myocardial work efficiency (GWE), defined as the percentage ratio of constructive work (GCW) to the sum of the GCW and wasted work (GWW), enable the analysis of the LV function independent from the afterload [10]. The normal values of the GWI and GCW vary from 1900–2100 mmHg% to 2200–2400 mmHg%. The reference value for the GWW is defined as 73 to 87 mmHg%, while the mean GWE is about 96% [19].

### 2.7. Statistical Analysis

The statistical analyses were performed using SPSS Statistics (version 24.0, IBM, Armonk, NY, USA). Kolmogorov–Smirnov was used to test for normal data distribution. The continuous variables are expressed as the mean ± standard deviation (SD) and the differences between the two groups were analyzed by Student’s *t*-test. The follow-up time was expressed by the median ± interquartile range. All the categorical variables were expressed as numbers and/or percentages. A Chi-squared or Fisher’s exact test was used to analyze the categorical variables as appropriate. The receiver operating characteristic (ROC) curve including the area under the curve (AUC) was used to evaluate the discrimination ability. A univariate Cox regression analysis was performed for age, sex, echocardiographic parameters, clinical symptoms and risk factors. Kaplan–Meier time-to-event analyses were performed and compared with the log-rank test. The kappa coefficient (κ) was used to assess the intra- and interobserver variability for the GWI measurements in 20 randomly selected patients. A *p*-value < 0.05 was considered to be statistically significant.

## 3. Results

The baseline demographic characteristics are shown in Table 1. Apart from arterial hypertension, which was more common in the group without AVR than in the group of patients with AVR (83% vs. 65%; *p* = 0.040), all the other parameters were not significantly different between the two groups (Table 1).

### 3.1. Echocardiographic Parameters

The guideline criteria, EOA and Vmax were similar in both groups, whereas the Pmean was significantly higher in the AVR group compared to the non-AVR group (Table 2). The left ventricular mass index was also higher in the AVR group (Table 2). The remaining echocardiographic parameters of LV function, right ventricular function and diastolic function were similar in both groups (Table 2).

### 3.2. Deformation Imaging

**Figure 1:**
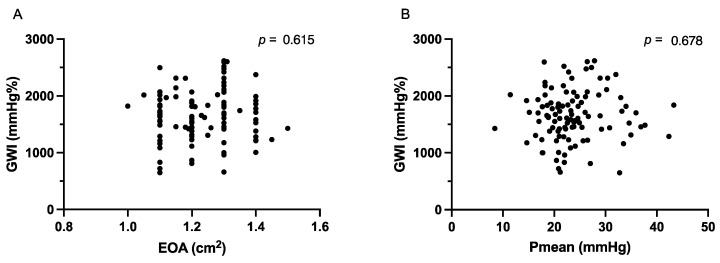
(**A**) Correlation of global work index (GWI) and effective orifice area (EOA) in all patients. (**B**) Correlation of GWI and mean transvalvular pressure gradient (Pmean) in all patients.

**Figure 2:**
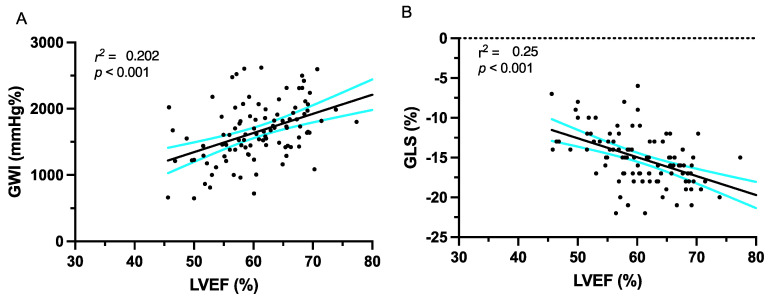
(**A**) Correlation of global work index (GWI) and left ventricular ejection fraction (LVEF). (**B**) Correlation between global longitudinal strain (GLS) and LVEF. The cyan lines represent the error bars around the interpolated black correlation line.

There were no significant differences between the parameters of the LV deformation between the group of patients with and without AVR (Table 3). Furthermore, no correlation was observed between the GWI and EOA or Pmean (Figure 1). In all the patients, the LVEF showed a weak correlation with the GWI and GLS (Figure 2).

### 3.3. Progression of AS Severity and Outcome

**Figure 3:**
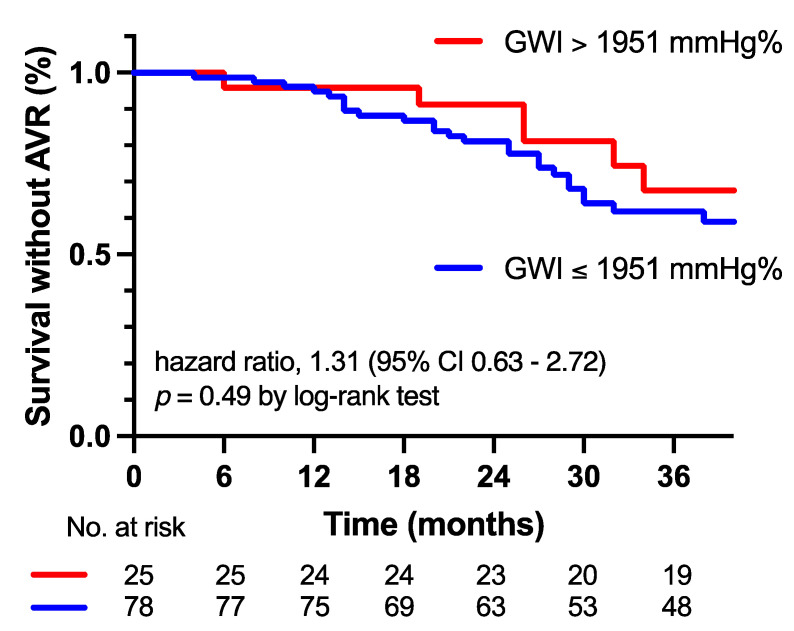
Kaplan–Meier estimates of survival without aortic valve replacement (AVR) according to global work index (GWI).

After a median follow-up of 30 ± 5 months, 37 patients (36%) were referred for an AVR. Survival without an AVR was 96% at 12 months and 80% at 30 months (>1951 mmHg%) versus 96% and 68% (≤1951 mmHg%). The prevalence of an AVR in patients with moderate AS with a GWI ≤ 1951 mmHg% and >1951 mmHg% was not significantly different, and a GWI ≤ 1951 mmHg% did not predict the need for an AVR (hazard ratio 1.31 (95% CI, 0.63–2.72), *p* = 0.49), displayed in Figure 3.

### 3.4. Sensitivity and Specificity of the Global Work Index

**Figure 4:**
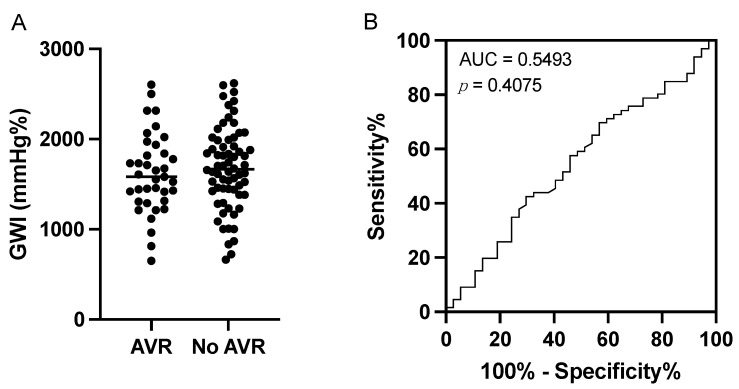
(**A**) Distribution of global work index (GWI) values between groups with and without the need for aortic valve replacement (AVR). (**B**) Receiver operator characteristics (ROC) curve analysis for GWI.

The sensitivity for a GWI ≤ 1951 mmHg% to predict the need for an AVR was 0.78, with a specificity of 0.26. There was no significant difference between the AVR group and non-AVR group. The distribution of the GWI values was very similar. Therefore, no specific cut-off value was found to discriminate between the AVR and non-AVR groups (Figure 4).

In the Cox regression analysis, the statistical significance could only be demonstrated for the mean gradient, so a multivariate analysis was not performed (Table 4).

### 3.5. Intra- and Interobserver Variability

The intra- (κ = 0.87; z = 4.53, *p* < 0.001) and interobserver (κ = 0.75; z = 4.33, *p* < 0.001) variability showed a high agreement for the GWI measurements in the 20 randomly selected patients. The intra- and interobserver variabilities for the remaining conventional echocardiographic measurements consistently showed good agreement as well.

## 4. Discussion

In this study, we aimed to investigate the prognostic value of the GWI in predicting the need for an AVR in patients with moderate AS defined by an EOA between 1.0 cm^2^ and 1.5 cm^2^.

The main findings of the present study are as follows:

(1) The GWI could not predict the need for an AVR in moderate AS patients.
(2) There was no difference in the incidence of clinical symptoms between the AVR and non-AVR groups.

The role of GLS in AS patients has already been discussed in previous studies. Lee et al. reported that GLS was associated with an increased risk of cardiac events in conservatively treated symptomatic severe AS patients [20]. Zhu et al. and Stassen et al. confirmed in patients with moderate AS that—while the LVEF is preserved—reduced LV GLS is associated with an increased risk of all-cause mortality [21,22]. The risk was even increased for AVR. In our study, there was no difference in the GLS between the AVR and non-AVR groups. The absence of a GLS difference between these groups may suggest a lack of subclinical myocardial damage, which has been linked to heart failure and diastolic dysfunction in previous studies [23].

Impaired GLS may reflect subclinical myocardial damage and increased tissue fibrosis as shown by Park et al., who studied 71 patients with severe AS using cardiac magnetic resonance and speckle-tracking imaging prior to an AVR in addition to histological examination by intraoperative endomyocardial biopsy [23]. The amount of fibrosis has been associated with lower GLS values [23] and has been shown to be a measure of LV systolic function that correlates with the parameters of myocardial contraction derived from pressure–volume loop analyses [24]. However, dynamic LV unloading experiments clearly demonstrated the dependence of GLS on different preload and afterload conditions [25,26]. Taking into account the LV loading conditions could improve the assessment of LV dysfunction at subclinical stages—especially in different types of LV hypertrophy [27,28,29]. Therefore, a reduced GWI implies possibilities to detect LV dysfunction in AS patients with reduced GLS at subclinical stages.

Urheim et al. showed that strain analyses by echocardiography can be used to construct pressure–volume loops non-invasively, but using invasive LV pressure measurements, and allow for the calculation of myocardial work [30]. In 2013, Russell et al. proposed the introduction of a normalized LV pressure curve, replacing the individual LV pressure by non-invasive brachial artery cuff pressure together with the combination of strain imaging and speckle-tracking echocardiography [8]. This study reported (1) a good correlation of the invasive and non-invasive LV pressure measurements and (2) of the pressure–strain loops and LV pressure. Furthermore, the regional pattern of glucose utilization (FDG-PET) was reflected in the regional LV pressure–strain loop pattern derived by using speckle tracking, which provides information on regional myocardial work [8]. Yet, the method by Russell et al. can be flawed by higher blood pressures. Therefore, a normalized pressure curve was obtained and used as a reference in the proposal of Russell et al. [8]. Based on the confirmation of these promising results, the method has been integrated into modern echocardiography software [9].

A recent study by Ilardi et al. [11] reported a significant difference in the GWI between asymptomatic moderate-to-severe AS patients who died during follow-up and those who did not (2603 ± 503 vs. 2307 ± 532 mmHg%). Ilardi et al. showed that a GWI ≤ 1951 mmHg% predicted all-cause and cardiovascular mortality at the 4-year follow-up [11]. In contrast, there was no difference in the GWI between patients who required a valve replacement and those who did not. For this reason, it was not possible to statistically define a cut-off value in our cohort. We therefore analyzed the cut-off value of 1951 mmHg%, which did not predict AVR in patients with moderate AS. As suggested by the similar graphical distribution of the GWI values in the AVR and non-AVR cohorts (Figure 4A), the ROC analysis did not reveal a specific cut-off value that could serve as both a sensitive and specific test to predict the need for an AVR. To determine a cut-off value, it is important to know the pre-test probability of needing an AVR, which is still difficult to determine. This means that even if the GWI is a good test for predicting prognosis in severe AS, the cut-off value is not universal and—as shown by the present analysis—should be determined for each patient and disease cohort.

In contrast to the study by Ilardi et al., only patients with moderate AS were included in the present study, whereas patients with severe AS were excluded. This difference can be explained by a lower mean EOA of 1.0 ± 0.35 cm^2^ in the present study compared to 1.24 ± 0.11 cm^2^ in Ilardi et al. [11].

In the study by Fortuni et al., the GWI showed an independent association with the New York Heart Association class heart failure symptoms (NYHA III and IV) in 120 patients with severe AS [12]. These results were not observed in our study, probably because most of the patients included in this study had no or only mild non-specific symptoms.

The conventional echocardiographic criteria, that are robust in identifying severe AS, are often less reliable in assessing the pathophysiological changes in moderate AS, as demonstrated by the limited correlation between the GWI and EOA or Pmean in our study. A possible explanation for these findings may lie in the complexity of LV loading in moderate AS. While the afterload is increased, the degree of hypertrophic response and LV adaptation may vary among patients [31]. The pathophysiological changes in moderate AS result in different remodeling patterns and adaptive responses. This heterogeneity could lead to variations in the GWI, which may not be adequately captured by a single cut-off value. However, in moderate AS, there may not be a well-defined cut-off value that accurately predicts clinical outcomes. The progression from moderate to severe AS is gradual and not exclusively determined by echocardiographic parameters, such as the EOA or Pmean. Therefore, applying a specific GWI cut-off value may not be appropriate in this cohort.

There may also be limitations in the measurement of the afterload: The accuracy of the GWI is highly dependent on the measurement of the afterload, which is approximated by brachial cuff blood pressure. In patients with moderate AS, arterial hypertension is common, with over 50% of patients experiencing high blood pressure [32]. The accuracy of estimating the LV pressure from the brachial artery pressure can be affected by several aspects such as arterial stiffness and calcification, potentially leading to inaccuracies in GWI calculations.

## 5. Limitations

We aimed to characterize a population of patients with moderate AS, but it cannot be excluded that pathophysiological changes (e.g., left ventricular hypertrophy) may have other causes (e.g., arterial hypertension). Due to the strict exclusion criteria, the sample size is limited, which may affect the ability of the study to detect significant correlations. The results of this cannot be extrapolated to all patients with AS. Death may be a competing event for an AVR, but those patients were excluded from our analysis.

## 6. Conclusions

The results of this study suggest that the GWI is not a reliable prognostic parameter for predicting the need for an AVR in patients with moderate AS. While the GWI has shown promise in patients with severe AS, its applicability in moderate AS remains uncertain. Further research is needed to improve the risk stratification and to identify predictors of clinical outcomes in patients with moderate AS.

## Figures and Tables

**Table 1:** Baseline demographic characteristics.

Variables	No Aortic Valve Replacement (n = 66)	Aortic Valve Replacement (n = 37)	*p*-Value
Age (years)	72.6 ± 10.7	71.4 ± 9.6	0.562
Male (%)	44 (67)	27 (73)	0.529
Weight (kg)	80.9 ± 14.6	83.6 ± 15.1	0.383
Height (cm)	170.3 ± 9.8	172.1 ± 8.0	0.315
BSA (m^2^)	1.95 ± 0.21	1.99 ± 0.20	0.342
BMI (kg/m^2^)	27.9 ± 4.3	28.0 ± 4.3	0.911
sBP (mmHg)	138.8 ± 13.7	141.3 ± 17.2	0.454
dBP (mmHg)	78.5 ± 10.5	79.3 ± 10.4	0.709
HR (1/min)	69.6 ± 10.2	71.0 ± 12.2	0.557
Arterial hypertension (%)	55 (83%)	24 (65%)	0.040
Diabetes mellitus (%)	9 (14%)	9 (24%)	0.203
Hypercholesterolemia (%)	30 (45%)	23 (62%)	0.100
COPD (%)	4 (6%)	4 (11%)	0.366
Atrial fibrillation (%)	12 (18%)	6 (16%)	0.798
CAD (%)	22 (33%)	15 (41%)	0.419
Stroke (%)	14 (21%)	3 (8%)	0.088
CKD ≥ 3 (%)	24 (36%)	14 (38%)	0.841
Smoker (%)	21 (32%)	9 (24%)	0.394
Dyspnea (%)	20 (30%)	9 (24%)	0.517
Angina pectoris (%)	11 (17%)	7 (19%)	0.799
Previous syncope (%)	2 (3%)	0 (0%)	0.289

BSA = body surface area; BMI = body mass index; sBP = systolic blood pressure; dBP = diastolic blood pressure; HR = heart rate; CAD = coronary artery disease; CKD = chronic kidney disease.

**Table 2:** Basic echocardiographic parameters.

Variables	No Aortic Valve Replacement (n = 66)	Aortic Valve Replacement (n = 37)	*p*-Value
EOA (cm^2^)	1.24 ± 0.11	1.23 ± 0.11	0.661
Vmax (m/s)	3.2 ± 0.4	3.3 ± 0.5	0.301
Pmean (mmHg)	22.7 ± 4.9	25.6 ± 7.5	**0.039**
LVEF (%)	60.8 ± 7.5	60.7 ± 7.2	0.947
TAPSE (mm)	20.8 ± 3.8	21.0 ± 3.6	0.791
E/E′	12.0 ± 4.8	13.5 ± 3.1	0.058
TR_Vmax_ (m/s)	28.9 ± 7.8	30.9 ± 7.2	0.193
LVMi (g/m^2^)	96.6 ± 35.1	113.4 ± 24.9	0.006

EOA = effective orifice area; Vmax = maximum transvalvular aortic flow velocity; Pmean = mean transvalvular pressure gradient; LVEF = left ventricular ejection fraction; TAPSE = tricuspid annular plane systolic excursion; TR_Vmax_ = maximum tricuspid regurgitation velocity; LVMi = left ventricular mass index.

**Table 3:** Parameters of left ventricular deformation.

Variables	No Aortic Valve Replacement (n = 66)	Aortic Valve Replacement (n = 37)	*p*-Value
GLS (%)	−15.1 ± 3.4	−15.0 ± 3.4	0.887
GWI (mmHg%)	1674 ± 456	1615 ± 441	0.522
GCW (mmHg%)	2103 ± 508	2096 ± 455	0.943
GWW (mmHg%)	221 ± 124	224 ± 177	0.928
GWE (%)	88.6 ± 5.9	88.8 ± 7.0	0.884

GLS = global longitudinal strain; GWI = global myocardial work index; GCW = global constructive work; GWW = global wasted work; GWE = global work efficiency.

**Table 4:** Univariable Cox proportional hazard model for aortic valve replacement in moderate aortic valve stenosis.

Variable	Hazard Ratio (95% Confidence Interval)	*p*-Value
Age (years)	1.0 (0.97–1.1)	0.765
Female sex	1.1 (0.45–2.5)	0.846
LVEF (%)	0.98 (0.94–1.0)	0.458
Pmean (mmHg)	1.1 (1.0–1.2)	0.015
EOA (cm^2^)	1.4 (0.039–55)	0.857
Hypertension	0.46 (0.21–1.0)	0.052
Diabetes mellitus	1.1 (0.46–2.6)	0.783
Dyspnea (≥NYHA II)	1.6 (0.60–4.0)	0.327
Angina	0.96 (0.33–2.5)	0.934

LVEF = left ventricular ejection fraction; Pmean = mean transvalvular pressure gradient; EOA = effective orifice area.

## Data Availability

The data sets used and/or analyzed during the current study are available from the corresponding author upon reasonable request.
